# Dynamic and Functional Characteristics of Predominant Species in Industrial Paocai as Revealed by Combined DGGE and Metagenomic Sequencing

**DOI:** 10.3389/fmicb.2018.02416

**Published:** 2018-10-09

**Authors:** Huipeng Liang, Huiying Chen, Chaofan Ji, Xinping Lin, Wenxue Zhang, Li Li

**Affiliations:** ^1^National Engineering Research Center of Seafood, School of Food Science and Technology, Dalian Polytechnic University, Dalian, China; ^2^Food Eco-engineering and Biotechnology Lab, College of Light Industry, Textile and Food Engineering, Sichuan University, Chengdu, China; ^3^College of Biotechnology Engineering, Sichuan University of Science and Engineering, Zigong, China

**Keywords:** industrial paocai, dominant species, functional characteristics, metagenomic sequencing, DGGE

## Abstract

The microbial community during the fermentation of industrial paocai, a lactic acid fermented vegetable food, was investigated via combined denaturing gradient gel electrophoresis (DGGE) and metagenomic sequencing. Firmicutes and Proteobacteria were identified as the dominant phyla during the fermentation. DGGE results of the bacterial community analysis showed that many genera were observed during the fermentation of industrial paocai, but the same predominant genus and species were observed: *Lactobacillus* and *Lactobacillus* (*L.*) *alimentarius*/*L. paralimentarius*. The abundance of *L. alimentarius*/*L. paralimentarius* increased fast during the initial stage of fermentation and approximately remained constant during the later stage. Metagenomic sequencing was used to finally identify the predominant species and their genetic functions. Metabolism was the primary functions of the microbial community in industrial paocai fermentation, including carbohydrate metabolism (CM), overview (OV), amino acid metabolism (AAM), nucleotide metabolism (NM), energy metabolism (EM), etc. The predominant species *L. alimentarius* and *L. paralimentarius* were involved in plenty of pathways in metabolism and played different roles in the metabolism of carbohydrate, amino acid, lipid to form flavor compounds during industrial paocai fermentation. This study provided valuable information about the predominant species in industrial paocai and its functional properties, which could enable us to advance our understanding of the fermentation mechanism during fermentation of industrial paocai. Our results will advance the understanding of the microbial roles in the industrial paocai fermentation and provide a theoretical basis for improving the quality of industrial paocai products.

## Introduction

Chinese paocai is a traditional lactic acid fermented vegetable food and could date back to 3,000 years ago (Xiong et al., 2012). It is generally served as a side dish and widely consumed in southwestern China. To date, the production of Chinese paocai is mostly based on spontaneous fermentation both in homemade and industrial processes. Nowadays, paocai continues to be made in many regions of China and has drawn an increasing attention in recent years, because it is rich in vitamins, probiotics, minerals, organic acids, and lactic acid bacteria (LAB) (Zhang Q. et al., 2016). Home-made paocai is made from assorted vegetables with the addition of seasonings, while industrial paocai was commonly made from a single type of vegetable without the addition of seasonings. After selecting and cleaning, raw vegetables (e.g., radish, cowpea, *Qingcai*, or *Zhacai*) were stacked with 8–15% (w/w) salt to withdraw the juice, and then spontaneously fermented based on the microbes present on raw materials under the ambient temperature. Similar to other fermented vegetables, LAB is often reported in paocai, including *Lactobacillus* (*L.*) *plantarum, L. casei, L. alimentarius, L. brevis, L. paracasei, L. pentosus, L. sakei, L. spicheri, L. zymae, L. hammesii, L. ginsenosidimutans, L. acetotolerans, Leuconostoc* (*Leu.*) *mesenteroides, Leu. Lactis, Enterococcus* (*Ent.*) *faecalis, Lactococcus* (*Lac.*) *lactis*, and *Pediococcus* (*Ped.*) *ethanolidurans* (Xiong et al., 2012; Yu et al., 2012; Cao et al., 2017). Although the species in different home-made paocai are dissimilar (Xiong et al., 2012; Cao et al., 2017), most of them belonged to genera *Lactobacillus, Leuconostoc, Pediococcus*, and *Enterococcus*. Many LAB species, such as *L. brevis* (Xia et al., 2017), *L. plantarum* (Liu et al., 2017), *Ent. faecium* (Liu et al., 2015), *L. pentosus*, and *Leu. mesenteroides* (Yan et al., 2008), are used as the starter in the production of home-made paocai to improve the quality. Nevertheless, there are limited reports on the microbial diversity of industrial paocai. The predominant species as well as their functions in industrial paocai remains to be fully characterized as the quality requirement of industrial production of paocai become increasingly higher.

As far as we know, culture-dependent methodology is often time-consuming and commonly understood yielding insufficient information about the microbial structure. For now, many powerful molecular ecological methods, such as denaturing gradient gel electrophoresis (DGGE), have extensively been used to explore the microbial community in the field of food (Shanqimuge et al., 2015; Zhang et al., 2015; Wang et al., 2016). In this study, DGGE was employed to identify the predominant microflora during the fermentation of industrial paocai. However, such analysis might not provide a complete profile of the microbial community and an accurate species identification because of its limitations and the 95% cut-off for sequence similarity. In the development of high-throughput sequencing, metagenomic sequencing has become a powerful methodology for the identification of species and the characterization of functions in food studies (Zhang J. et al., 2016; Escobar-Zepeda et al., 2016). Then in this study metagenomic sequencing was employed to determine the predominant species and decipher their gene functions in industrial paocai fermentation.

The major aim of this work, therefore, was to investigate the dominant microbial community at species level during the fermentation of the industrial paocai, and characterize their gene functions by combined DGGE and metagenomic sequencing. Our results will contribute to understand and improve the microbial fermentation process of industrial paocai.

## Materials and Methods

### Sample Collection and Physicochemical Properties

Twenty brine samples of industrial paocai in different fermentation stage, including *Qingcai* paocai (QP) and *Zhacai* paocai (ZP), were collected from a famous paocai factory which is located in Meishan, Sichuan Province of China, in 2016. The samples were kept on ice and transported to our laboratory. A pH meter (PHS-3C, China) was used to determine the pH values. Total titratable acidity (TTA) was determined according to the titration method of AOAC 942.15. Salinity of samples was determined via a digital salt meter (ATAGO, Japan). The nitrite content was determined using the GB/T 5009.33-2010 method and shown in units of mg/kg.

### DNA Extraction and PCR Amplification

Total genomic DNA was extracted from ten milliliters homogenized paocai brine samples using E.Z.N.A.^®^ DNA Kit (Omega, United States) following the manufacturer’s instructions and then stored at -20 °C until use. The highly variable V3 region of bacterial 16S ribosomal RNA (rRNA) gene was amplified by using 357F and 517R primers (Muyzer et al., 1993). Reactions (50 μL) included 2 × PCR Mix (TIANGEN Biotech, China), 20 pmol primers, DNA templates and distilled water. The PCR program for the 16S rRNA gene was performed as described previously in a MyCycler^TM^ Thermal (Bio-Rad, United States) (Liang et al., 2016a). For DGGE analysis, a 40 nucleotide GC-clamp were appended to the 5^′^ end of the forward primer (Muyzer et al., 1993). All the amplification products were checked on 2% agarose gels.

### DGGE Analysis and Band Sequencing

Amplified products were subsequently subjected to DGGE using a D-Code^TM^ Universal Mutation Detection System (Bio-Rad, United States). An aliquot of 20 μL of each amplified product was loaded onto the gel. Electrophoresis was carried out with 8% polyacrylamide gels in 1× TAE buffer at 60°C with a linear gradient of 30–55% denaturant [100% corresponds to 7 M urea and 40% (v/v) formamide] for the bacterial community at constant voltage of 200 V for 4 h at 60°C, respectively. Subsequently, the gels were stained for 45 min with SYBR Green I (1:10,000 v/v) and visualized using Gel Doc^TM^ XR (Bio-Rad, United States). The major bands were excised and the eluted DNA was re-amplified as described above using the primer without GC-clamp. The PCR products were purified and then delivered to a biotech company (Sangon, Shanghai, China) for clone sequencing. The sequence information was achieved by aligning the results with the sequences in GenBank using BLAST ^1^.

### Metagenomic Sequencing Analysis

Genomic DNA extracted from the samples was prepared for sequencing using E.Z.N.A^TM^ Mag-Bind Soil DNA Kit according to the manufacturer’s protocol. An Illumina library was prepared from total using NEBNext^®^ Ultra^TM^ DNA Library Prep Kit for Illumina^®^ (Illumina, San Diego, CA, United States) following the manufacturer’s specifications with an average fragment size of 500 base pair (bp). Agencourt AMPure XP Kit (Beckman, CA, United States) was used to purify the amplicons of DNA in libraries. The sequencing (2 × 150 bp) was performed on the HiSeq 2500 platform (Illumina, Inc., San Diego, CA, United States). Quality control was performed using Trimmomatic v0.36 (Bolger et al., 2014). Sequences containing N base and adapters or with a low quality value (Q value less than 20) were removed. *De novo* assembly was performed using IDBA-UD v1.1.1 (Peng et al., 2012) with an optimal k-mer parameters. Open reading frame (ORF) prediction was performed by running Prodigal software v2.60 (Liu et al., 2013) on assembled sequences and translated into protein sequences. The predicted sequences were clustered (with 95% identities and 90% coverage) and the longest sequence was selected in each cluster to construct the non-redundant gene catalog by CD-HIT v4.6 (Li and Godzik, 2006). The assembled unigenes were searched against the NR (NCBI non-redundant protein sequences)^2^ database using DIAMOND (Buchfink et al., 2015), the Kyoto Encyclopedia of Genes and Genomes database (KEGG)^3^ using GhostKOALA v0.9.7 (Kanehisa et al., 2016).

### Data Analysis

The number, migration, and intensity of DGGE bands were analyzed via Quantity One (Bio-Rad, United States). The Shannon and Pielou’s index were determined based on the relative quantity of the DGGE bands (Shannon, 1949; Pielou, 1966). SIMCA-P ver. 13.0 software (Umetrics, Sweden) enabled principal component analysis (PCA) based on the relative abundance of microbiota during the fermentation of industrial paocai. Statistical analysis was performed using the SPSS (SPSS Inc., United States). Heat maps showed the microbial distribution at different classification levels and were constructed using the HemI Software (Heatmap Illustrator, version 1.0).

## Results

### Physicochemical Properties During the Fermentation of Industrial Paocai

**FIGURE 1:**
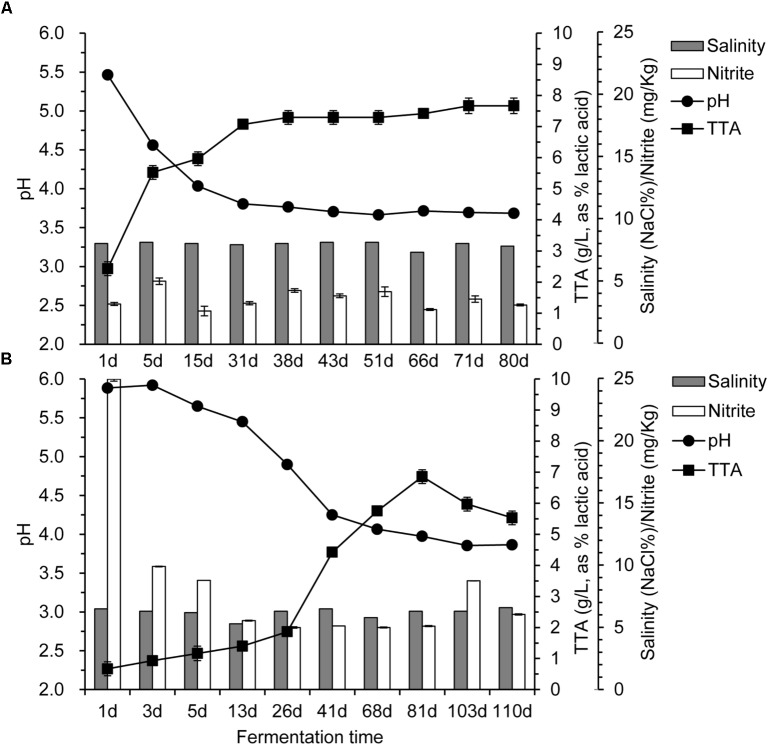
Changes in pH, total titratable acidity (TTA), salinity, and nitrite during industrial paocai fermentation. Panels **(A,B)** represent industrial ZP and QP, respectively.

The changes of pH, TTA, salinity, and nitrite during fermentation of the industrial paocai are shown in **Figure 1**. The pH declined before 38 d, and then remained stable until the end of fermentation. TTA values were increased with different speeds for ZP and QP, and then remained stable during the later stage of fermentation. But the content of TTA in ZP was greater than that in QP (**Figure 1**). The salinity of ZP and QP was almost unchanged during the fermentation (**Figure 1**). The salinity of ZP was about 8.0, while that of QP was about 6.5. The nitrite content of ZP was almost unchanged (**Figure 1A**), while that of QP was decreased during the fermentation process (**Figure 1B**). But the nitrite content of ZP and QP during the later fermentation stage was significantly lower than the national health standard’s safe level (20 mg/kg).

### Bacterial Community During Fermentation Revealed by DGGE

**FIGURE 2:**
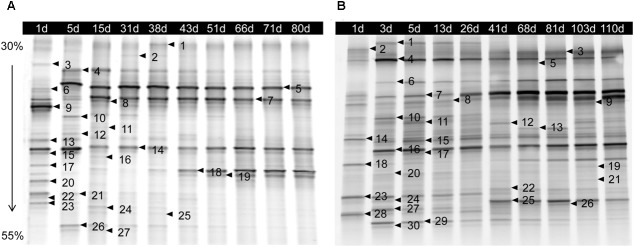
DGGE profile of microbiota from microbial DNA extracted from industrial ZP **(A)** and QP **(B)** samples collected over fermentation process. The denaturing gradient was 30–55%. The bands indicated by the arrows and numbers were sequenced and the alignment results are listed in **Table 1**.

**FIGURE 3:**
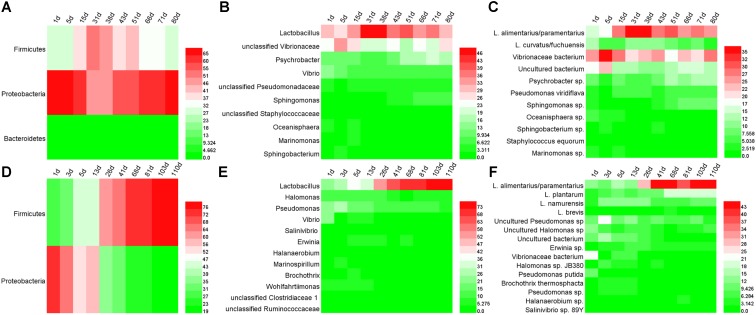
Microbial distributions in industrial paocai during fermentation. Panels **(A–F)** represent the heatmaps of bacterial community in industrial ZP (QP) at phylum, genus, and species level.

The bacterial DGGE profile of ZP and QP is illustrated in **Figure 2**. In the DGGE profile of ZP, 27 bands were identified (**Table 1**) and fell into three phyla: Firmicutes, Proteobacteria, and Bacteroidetes (**Figure 3A**), but more than 96% of the abundance were assigned to the phylum Firmicutes and Proteobacteria. A total of 10 genera, such as *Lactobacillus, Psychrobacter, Vibrio, Sphingomonas, Oceanisphaera, Sphingobacterium, Staphylococcus, Marinomonas*, etc., were detected in ZP (**Figure 3B**). The relative abundance of *Lactobacillus* increased at first and then decreased to a stable level (about 30%) during the fermentation, while that of some genera, such as *Vibrio, Oceanisphaera, Marinomonas*, and *Sphingomonas*, showed the reverse changes (**Figure 3B**). At species level, the dominant band was identified as *L. alimentarius* or *L. paralimentarius* (**Figure 3C**). Two phyla, including Firmicutes and Proteobacteria, were observed in QP (**Figure 3D**). Therefore, Firmicutes and Proteobacteria were prevailing during the fermentation of industrial paocai. Compared with ZC paocai, a total of 12 genera, such as *Lactobacillus, Halomonas, Pseudomonas, Vibrio, Salinivibrio, Erwinia, Halanaerobium*, etc., were observed in industrial QP (**Figure 3E**). The abundance of *Lactobacillus*, including *L. alimentarius*/*paralimentarius, L. plantarum, L. namurensis*, and *L. brevis*, increased during the entire fermentation of QP and then remained at about 70% during the later fermentation stage (**Figures 3E,F**). Therefore, *Lactobacillus* and *L. alimentarius*/*paralimentarius* was predominant genus and species during the fermentation process of industrial paocai.

**Table 1:** Sequencing results of selected DGGE bands from the DGGE profiles.

Bands ^a^		Closest relative ^b^	Identity (%)	Length (bp)	Accession no.
ZP	1,2,4,11,13,20,26	Vibrionaceae bacterium	98,99	196	KT356841.1
	3,6,14,16	Uncultured bacterium	100	196	LC140853.1
	5,7,17,24	*Lactobacillus alimentarius/Lactobacillus paralimentarius*	99,100	196	AB919114.1/MF942368.1
	8	*Pseudomonas viridiflava*	100	196	KX640928.1
	9,23	*Lactobacillus curvatus/Lactobacillus fuchuensis*	100	196	LC130556.1/LC130557.1
	10	*Psychrobacter* sp.	99	195	KU198795.1
	12	*Sphingobacterium* sp.	100	191	KX527636.1
	15	Uncultured bacterium	100	183	KJ475839.1
	18	*Psychrobacter* sp.	100	196	KX214117.1
	19	*Sphingomonas* sp.	100	164	KR856419.1
	21	*Oceanisphaera* sp.	98	196	HM566011.1
	22	*Marinomonas* sp.	99	196	KM979047.1
	25	*Staphylococcus equorum*	99	196	KX495495.1
	27	Uncultured bacterium	99	195	EU465483.1
QP	1,3,7,9,12,18,19,25	*Lactobacillus alimentarius/Lactobacillus paralimentarius*	99,100	192-197	AB919114.1/MF942368.1
	2	Vibrionaceae bacterium	99	196	KJ158197.1
	4	Uncultured *Pseudomonas* sp.	100	196	JN873217.1
	5,6,8,30	*Lactobacillus plantarum*	99,100	196	KX519704.1
	10	*Pseudomonas* sp.	100	196	KX389675.1
	11	*Brochothrix thermosphacta*	96	195	KT767854.1
	13	*Halanaerobium* sp.	100	196	KR612329.1
	14	*Pseudomonas putida*	100	195	KX436994.1
	15	*Erwinia* sp.	99	196	LK054598.1
	16,24	*Lactobacillus namurensis*	99	196	KT285577.1
	17,28	Uncultured *Halomonas* sp.	99	187,196	LC140852.1
	20	Uncultured bacterium	99	172	AB818639.1
	21	Uncultured bacterium	97	171	LN849544.1
	22	*Salinivibrio* sp. 89Y	100	196	KP795377.1
	23	Uncultured bacterium	98	197	KC208464.1
	26	*Lactobacillus brevis*	100	196	KX519532.1
	27	Uncultured bacterium	99	196	LC140859.1
	29	*Halomonas* sp. JB380	100	197	KF669533.1

*^*a*^ZP and QP represented the industrial *Zhacai* and *Qingcai* paocai. Each number corresponds to the bands indicated in **Figure 2**. ^*b*^Sequences were compared with those in NCBI database*.

### Multivariate Analysis of DGGE Profiles

**FIGURE 4:**
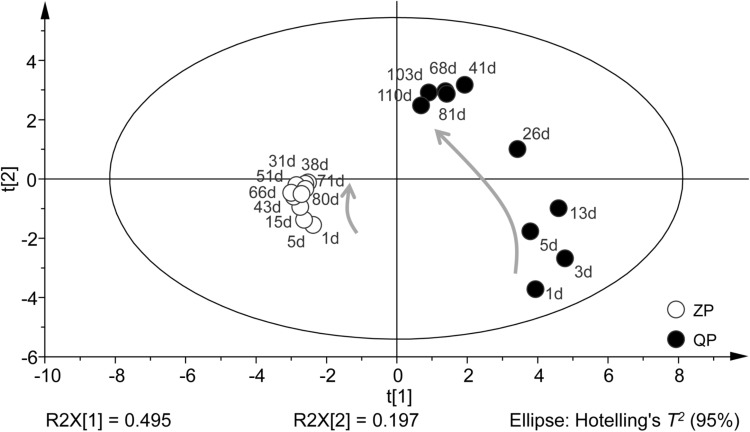
PCA of bacterial distribution in the industrial ZP and QP during fermentation based on the DGGE data of bacterial community. Samples are displayed by circles. The directions of the curved arrows indicate the routes of data points on the score plots during fermentation. The elliptical area represents 95% confidence intervals.

The profiles of bacterial community during the fermentation of industrial paocai obtained by DGGE were statistically analyzed statistically via PCA. PCA analysis based on DGGE data of bacterial community during the fermentation of industrial ZP and QP was basically similar and the two axes explained 49.50 and 19.70% of the variation in bacterial community differentiation, respectively (**Figure 4**). The elliptical area on the PCA plot represents 95% confidence intervals of the modeled variation analyzed by PCA-Hotelling’s *T^2^* test and all samples were in the elliptical area, which indicated that there were no outliers in the data. In the results of PCA analysis, the bacterial community structures in ZP and QP were different, but their variation tendency was similar (**Figure 4**).

### Determination of Predominant Species by Metagenomic Sequencing

**FIGURE 5:**
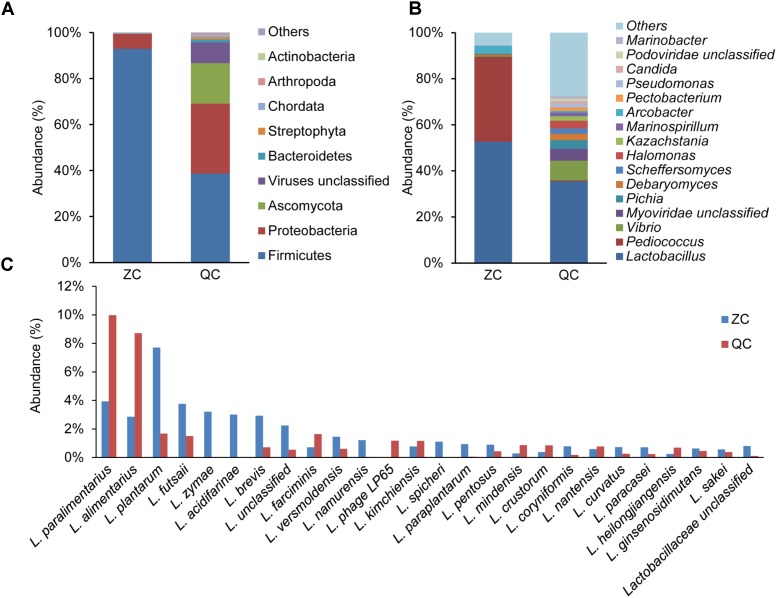
Taxonomy of the microbial community in matured industrial ZP and QP using metagenomic sequencing. The phylum with abundance ≥0.1% **(A)** and the genus with abundance ≥0.1% **(B)** in at least one sample were presented. Only the species affiliated with Lactobacillus with abundance ≥0.5% **(C)** in at least one sample was presented. L, *Lactobacillus*.

To determine the dominant species in industrial ZP and QP, metagenomic sequencing was employed to analyze the microbial community in matured industrial ZP and QP. Sequencing and assembly statistics are presented in **Supplementary Table S1**. After gene prediction and construction, a total of 94 phyla, including Firmicutes, Proteobacteria, Ascomycota, Viruses unclassified, Bacteroidetes, Streptophyta, Chordata, Arthropoda, and Actinobacteria, was identified in industrial ZP and QP, and two phyla, including Firmicutes (92.97 and 38.67%) and Proteobacteria (6.51 and 30.41%), were detected with a high abundance (**Supplementary Figure S1**). A total of 2174 genera were detected and *Lactobacillus* (52.66 and 35.41%) was detected as predominant genus in industrial ZP and QP (**Figure 5**). *L. paralimentarius, L. alimentarius, L. plantarum, L. futsaii, L. zymae, L. acidifarinae, L. brevis*, and *L. farciminis* were observed as the shared species in ZP and QP with a higher abundance (**Figure 5**).

### Functional Characterization of Predominant Species in Industrial Paocai

**FIGURE 6:**
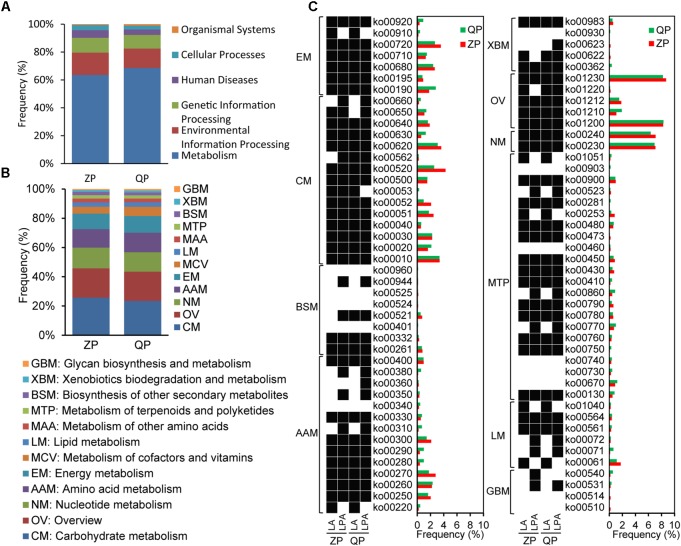
Gene functional annotation **(A)** of microbial community, KEGG pathways of metabolism **(B)**, and functional roles of *L. alimentarius* and *L. paralimentarius* in metabolism **(C)** in industrial ZP and QP. **(C)** The pathways with abundance ≥0.1% in at least one sample were presented. The black box represented genes encoding related enzymes in the pathway were detected while white box represented not. LA, *L. alimentarius* and LPA, *L. paralimentarius*.

According to the blast KEGG, the functional profiles of microbial genes in industrial ZP and QP were classified into metabolism, environmental information processing, genetic information processing, human diseases, cellular processes, and organismal systems (**Figure 6A**). Among them, metabolism was noted with the highest abundance. Carbohydrate metabolism (CM), overview (OV), amino acid metabolism (AAM), nucleotide metabolism (NM), and energy metabolism (EM) were observed with a high abundance (≥10%) in metabolism (**Figure 6B**). In them, the main KEGG Orthology (KO) entries with an abundance of above 0.1% were presented in **Figure 6C**. The identified enzymes produced by *L. paralimentarius* and *L. alimentarius* in these KO entries (with a high abundance ≥10%) are shown in **Supplementary Table S2**. Based on the identified enzyme types, *L. paralimentarius* and *L. alimentarius* were involved in plenty of metabolic pathways in industrial paocai, such as glycolysis/gluconeogenesis (ko00010), carbon metabolism (ko01200), biosynthesis of amino acids (ko01230), glycine, serine, and threonine metabolism (ko00260), etc. (**Figure 6C**).

## Discussion

To some extent, the spontaneous fermentation of vegetable depends on the microorganisms presented on the surface of raw materials, which leads to the proliferation of various dominant microorganisms. This made it difficult to produce the industrial paocai with uniform and good quality. Therefore, the investigation of microbial communities in industrial paocai is tremendously beneficial to achieve a greater understanding of spontaneous industrial paocai fermentation processes. In the present work, Firmicutes and Proteobacteria were observed as the dominant bacterial phylum in industrial paocai during fermentation processes (**Figures 3A,D**, **5A**). The same dominant phylum was also observed in paocai samples (Liang et al., 2016b; Yang et al., 2018; Wang and Shao, 2018). The microbial community structure detected in industrial ZC and QC paocai was different (**Figures 3**, **5**). Remarkably, amongst the genera detected in industrial ZC and QC paocai, *Lactobacillus* was predominant during the fermentation. This was in agreement with previous studies (Liang et al., 2016b, 2018a,b; Yang et al., 2018; Wang and Shao, 2018). The genus of *Lactobacillus*, which could produce plenty of acids, was always reported as the predominant LAB in fermented vegetables by culture-dependent or culture-independent methods (Yu et al., 2012; Xiong et al., 2012; Cao et al., 2017; Zhang et al., 2018). Xiao et al. (2018) found that *Lactobacillus, Leuconostoc, Achromobacter*, and *Pediococcus* were closely correlated with flavors in Chinese Sichuan paocai by using high-throughput sequencing and flavor analyses. Therefore, the metabolism of *Lactobacillus* during the paocai fermentation process potentially contributed to the formation of flavor. The genus of *Vibrio* was also detected in industrial paocai in this study. It is further detected in other fermented vegetables (like salted cabbage and kimchi) and might have originated from salt (Park et al., 2012; Hong et al., 2014; Lee et al., 2017). *Psychrobacter* can tolerate a wide range of salt concentrations and produce lipases (Bowman, 2006). Some *Pseudomonas* species have been reported to produce esters, ketones, hydrocarbons, alcohol, and sulfur compounds (Tamsyn et al., 2018). Therefore, these genera may contribute to the formation of flavor during fermentation of industrial paocai. In addition, there were also some other genus observed in this study and all of them could be found in previous studies on paocai (Liang et al., 2016a, 2018a,b; Cao et al., 2017; Xiao et al., 2018).

All in all, the compositions of microbial phyla detected in industrial ZC and QC paocai were same, while that of genera was different. These may be primarily due to the different raw materials were used. But the same predominant genus *Lactobacillus* during industrial ZC and QC paocai fermentation was identified. In fermented vegetables, many species affiliated with *Lactobacillus* were often observed as the dominant microorganism. Then they were used as the starter for inoculation fermentation to obtain a good quality product or ensure its safety in paocai production (Yan et al., 2008; Xiong et al., 2014; Liu et al., 2017; Xia et al., 2017; Du et al., 2018). *L. plantarum, L. casei*, and *L. zeae* were detected as dominant strains during Chinese sauerkraut fermentation by culture-dependent method (Xiong et al., 2012). *L. acetotolerans* and *L. brevis* were observed as the dominant species in homemade Sichuan paocai brines by PacBio SMRT sequencing technology (Cao et al., 2017). *L. acetotolerans, L. brevis*, and *L. buchneri* were the major species present in Chongqing radish paocai brine using PacBio SMRT sequencing (Yang et al., 2018). In this study, *L. alimentarius* and *L. paralimentarius* was observed for the first time as the predominant species by DGGE during the fermentation of industrial paocai (**Figures 3C,F**, **5C**). Potentially, *L. alimentarius* or *L. paralimentarius* could be used as a starter for the production of paocai by inoculated fermentation in industrial production. There was a significant variance in the most dominant bacterial species present in fermented vegetables. The production process, raw materials and geographical distribution could account for this difference (Wang and Shao, 2018; Yang et al., 2018; Zhang et al., 2018).

*Lactobacillus alimentarius* and *L. paralimentarius* were detected as the predominant species by DGGE during the fermentation of industrial ZC and QC paocai. In our previous study on industrial QP, the same potential dominant species during fermentation were also observed using DGGE (Liang et al., 2018b). But the length of the fragment to be resolved by DGGE is limited. This represents a limiting resolution and makes it difficult to achieve a reliable identification of the microbial species within the same genus (Kisand and Wikner, 2003; Ercolini, 2004). Then a single band may be composed of several species. For example, a bacterial DGGE band was identified as *L. alimentarius* or *L. paralimentarius*. Therefore, metagenomic sequencing was employed to determine accurate species in industrial paocai. In metagenomic sequencing analysis, Firmicutes and Proteobacteria were predominant phyla in industrial paocai (**Figure 5A**). *L. paralimentarius, L. alimentarius*, and *L. plantarum* were observed as the predominant species in industrial ZC and QC paocai (**Figure 5C**). This was in accordance with the DGGE results.

Metagenomic analysis of the industrial paocai samples made it possible to reveal not only microbial community structure at the species level but also the metabolic potential in industrial paocai. The species related to CM in industrial ZP were reported in our previous study (Liang et al., 2018a). But the gene functions of dominant species in industrial ZC and QC paocai remain unknown. It is generally accepted that microorganism, especially LAB, contributes significantly to the properties of various fermented foods, where they contribute to the flavor, texture and nutrition (Gaspar et al., 2013; Douillard and de Vos, 2014; Xiao et al., 2018). The microbial metabolism of carbohydrate, lipids, and protein produce the complex compounds which form the sensory properties of fermented food. In industrial paocai, metabolism was primary functions of the microbial community (**Figure 6A**). CM, OV, AAM, NM, and EM were the main pathways in metabolism (**Figure 6B**). In this study, the predominant species, namely, *L. alimentarius* and *L. paralimentarius* were involved in plenty of pathways, especially these main pathways in metabolism (**Figure 6C**). Among these observed pathways, CM was the main metabolism in industrial paocai. *L. alimentarius* and *L. paralimentarius* took part in many entries in the CM, such as glycolysis/gluconeogenesis (ko00010), pyruvate metabolism (ko00620), amino sugar and nucleotide sugar metabolism (ko00520), fructose and mannose metabolism (ko00051), and starch and sucrose metabolism (ko00500), producing some acids, aldehydes, and alcohols through producing multiple enzymes (**Supplementary Table S2**). *L. alimentarius* was reported to be able to degrade nitrite (Tang et al., 2016). In this study, the present of *L. alimentarius* might be the reason for lower nitrite content during the later stage of fermentation (**Figure 1**). In the entries of OV, the predominant species were involved in carbon metabolism (ko01200), biosynthesis of amino acids (ko01230), 2-oxocarboxylic acid metabolism (ko01210), fatty acid metabolism (ko01212), and degradation of aromatic compounds (ko01220), in which various flavor compounds are generated. AAM is important in food quality because amino acids, especially, sulfuric, aromatic, and the branched-chain amino acid, are precursors of many flavor compounds (Fernandez and Zuniga, 2006). *L. alimentarius* and *L. paralimentarius* were involved in cysteine and methionine metabolism (ko00270), tryptophan metabolism (ko00380), tyrosine metabolism (ko00350), phenylalanine metabolism (ko00360), and so on in this study (**Figure 6C**). Potentially, the amino acids are converted to various acids, alcohols, aldehydes, esters, and sulfur compounds for specific flavor development of industrial paocai. Lipids are also the important source of flavor compounds. *L. alimentarius* and *L. paralimentarius* were also involved in the fatty acid degradation and biosynthesis (ko00061 and ko00071), glycerophospholipid metabolism (ko00564), glycerolipid metabolism (ko00561), biosynthesis of unsaturated fatty acids (ko01040), and synthesis and degradation of ketone bodies (ko00072) (**Figure 6C**). In addition, the other metabolisms related to predominant species, such as biosynthesis of other secondary metabolites and metabolism of terpenoids and polyketides, also contribute to the flavor in industrial paocai. But the correlation between metabolic functions of the predominant species and the formation of flavor deserves further research.

## Conclusion

In the present study, the microbial community during the fermentation of industrial paocai was revealed and the predominant species were identified via combined DGGE and metagenomic sequencing. In the results of bacterial analysis, Firmicutes and Proteobacteria were the predominant bacterial phylum during the fermentation of industrial paocai. Plenty of different genera were observed, but the same predominant genus was obtained during the fermentation of industrial paocai. The abundance of *Lactobacillus* increased at first and remained stable during the later fermentation stage of industrial paocai. *L. paralimentarius* and *L. alimentarius* were the predominant species during fermentation. Amongst all the functional profiles, metabolism was the primary function of the microbial community in industrial paocai, including CM, OV, AAM, NM, EM, and so on. The predominant species were involved in plenty of pathways in metabolism and played different roles in the formation of flavor during the fermentation of industrial paocai. The investigation of the structures and gene functions at the species level in this study can partially explain the potential roles of the predominant population in the fermentation process of industrial paocai.

## Author Contributions

HL designed the experiment, analyzed the metagenomic sequence, and wrote the paper. HC determined the physicochemical properties. CJ analyzed the data obtained from metagenomic sequencing analysis. XL performed PCR-DGGE and its analysis. WZ revised the paper. LL performed sampling of the data.

## Conflict of Interest Statement

The authors declare that the research was conducted in the absence of any commercial or financial relationships that could be construed as a potential conflict of interest.

## Footnotes

1 www.ncbi.nlm.nih.gov/BLAST/

2 http://ncbi.nlm.nih.gov/

3 http://www.kegg.jp
