# Supplementary material for: Dynamic and Functional Characteristics of Predominant Species in Industrial Paocai as Revealed by Combined DGGE and Metagenomic Sequencing
Source: Front Microbiol. 2018 Oct 9;9:2416. doi: 10.3389/fmicb.2018.02416 (PMC6189446; doi:10.3389/fmicb.2018.02416)
Supplement: Supplementary file 1 [file Table_1.docx]

**Supplementary Table S1** Sequence statistics for the industrial ZP and QP comparing raw and high quality sequence data used in downstream analysis.

|  | ZP | | QP | |
| --- | --- | --- | --- | --- |
| **data pre-processing** | Raw | Quality Control | Raw | Quality Control |
| Total Reads Count (#) | 52139340 | 42991100 | 54202834 | 49290812 |
| Total Bases Count (bp) | 7820901000 | 6083796645 | 8130425100 | 7026029255 |
| Average Read Length (bp) | 150 | 141.51 | 150 | 142.54 |
| GC Bases Count (bp) | 3136319397 | 2423529019 | 3563114556 | 3069158945 |
| GC Bases Ratio (%) | 40.10 | 39.84 | 43.82% | 43.68% |
| **Assembly** | Contigs statistics | | Contigs statistics | |
| No.(#) | 70000 | | 270124 | |
| N50 (bp) | 1764 | | 1439 | |
| Total length (bp) | 71034340 | | 267309683 | |
| Average length (bp) | 1014.78 | | 989.58 | |
| GC content (%) | 42.90 | | 45.74 | |
| Max length (bp) | 138773 | | 298431 | |
| **Contigs** **statistics** | Gene  prediction | UniGene  construction | Gene  prediction | UniGene  construction |
| No.(#) | 113742 | 100120 | 417621 | 396250 |
| N50 (bp) | 702 | 741 | 678 | 696 |
| Total length (bp) | 60615984 | 57308760 | 223735170 | 216758775 |
| Average length (bp) | 532.93 | 572.40 | 535.74 | 547.03 |
| GC content (%) | 43.63 | 43.89 | 46.89 | 46.99 |
| Max length (bp) | 32421 | 32421 | 15075 | 15075 |
| Min length (bp) | 102 | 102 | 102 | 102 |
| ≥ 500 bp | 43258 | 42776 | 152101 | 149419 |
| ≥ 1000 bp | 12916 | 12884 | 45695 | 45360 |

Abbreviations: ZP, *Zhacai* paocai; QP, *Qingcai* paocai; bp, base pairs.
