# Supplementary material for: Dynamic and Functional Characteristics of Predominant Species in Industrial Paocai as Revealed by Combined DGGE and Metagenomic Sequencing
Source: Front Microbiol. 2018 Oct 9;9:2416. doi: 10.3389/fmicb.2018.02416 (PMC6189446; doi:10.3389/fmicb.2018.02416)
Supplement: Supplementary file 2 [file Table_2.docx]

**Supplementary Table S2** Functions of enzymes ecoded by predominant species in the industrial ZP and QP.

| KO ID | Function | Pathway  A* | Pathway  B | ZP | | QP | |
| --- | --- | --- | --- | --- | --- | --- | --- |
|  |  |  |  | LA | LPA | LA | LPA |
| K00014 | aroE [EC:1.1.1.25] | AAM | ko00400 | + | + | + | + |
| K00055 | E1.1.1.90 [EC:1.1.1.90] | AAM | ko00350,ko00360 | - | + | - | - |
| K00058 | serA, PHGDH [EC:1.1.1.95] | AAM | ko00260 | - | + | - | + |
| K00135 | gabD [EC:1.2.1.16 1.2.1.79 1.2.1.20] | AAM | ko00250,ko00310,ko00350 | - | + | - | + |
| K00147 | proA [EC:1.2.1.41] | AAM | ko00330 | - | + | - | + |
| K00215 | dapB [EC:1.17.1.8] | AAM | ko00300 | + | - | + | - |
| K00259 | ald [EC:1.4.1.1] | AAM | ko00250 | - | + | - | + |
| K00262 | E1.4.1.4, gdhA [EC:1.4.1.4] | AAM | ko00220,ko00250 | + | - | + | - |
| K00286 | proC [EC:1.5.1.2] | AAM | ko00330 | + | - | + | - |
| K00547 | mmuM, BHMT2 [EC:2.1.1.10] | AAM | ko00270 | - | - | + | - |
| K00600 | glyA, SHMT [EC:2.1.2.1] | AAM | ko00260 | - | + | - | + |
| K00609 | pyrB, PYR2 [EC:2.1.3.2] | AAM | ko00250 | - | + | - | - |
| K00626 | E2.3.1.9, atoB [EC:2.3.1.9] | AAM | ko00280,ko00310,  ko00380 | - | + | - | + |
| K00651 | metA [EC:2.3.1.46] | AAM | ko00270 | + | + | + | - |
| K00764 | purF, PPAT [EC:2.4.2.14] | AAM | ko00250 | + | - | + | - |
| K00789 | metK [EC:2.5.1.6] | AAM | ko00270 | - | - | - | + |
| K00800 | aroA [EC:2.5.1.19] | AAM | ko00400 | - | + | - | + |
| K00826 | E2.6.1.42, ilvE [EC:2.6.1.42] | AAM | ko00270,ko00280,  ko00290 | + | - | + | - |
| K00841 | patA [EC:2.6.1.-] | AAM | ko00300 | - | + | - | + |
| K00865 | glxK, garK [EC:2.7.1.165] | AAM | ko00260 | + | + | - | + |
| K00928 | lysC [EC:2.7.2.4] | AAM | ko00260,ko00270,  ko00300 | - | + | + | + |
| K00931 | proB [EC:2.7.2.11] | AAM | ko00330 | + | - | + | - |
| K01243 | mtnN, mtn, pfs [EC:3.2.2.9] | AAM | ko00270 | - | + | - | + |
| K01439 | dapE [EC:3.5.1.18] | AAM | ko00300 | - | + | - | + |
| K01485 | codA [EC:3.5.4.1] | AAM | ko00330 | - | - | + | - |
| K01581 | E4.1.1.17, ODC1, speC, speF [EC:4.1.1.17] | AAM | ko00330 | - | - | - | + |
| K01586 | lysA [EC:4.1.1.20] | AAM | ko00300 | + | - | + | - |
| K01641 | E2.3.3.10 [EC:2.3.3.10] | AAM | ko00280 | - | + | - | + |
| K01652 | E2.2.1.6L, ilvB, ilvG, ilvI [EC:2.2.1.6] | AAM | ko00290 | - | + | - | + |
| K01738 | cysK [EC:2.5.1.47] | AAM | ko00270 | - | + | - | + |
| K01756 | purB, ADSL [EC:4.3.2.2] | AAM | ko00250 | - | + | - | + |
| K01779 | racD [EC:5.1.1.13] | AAM | ko00250 | + | - | + | - |
| K01834 | PGAM, gpmA [EC:5.4.2.11] | AAM | ko00260 | + | - | + | - |
| K01928 | murE [EC:6.3.2.13] | AAM | ko00300 | - | + | - | + |
| K01929 | murF [EC:6.3.2.10] | AAM | ko00300 | - | + | - | + |
| K01939 | purA, ADSS [EC:6.3.4.4] | AAM | ko00250 | - | + | - | + |
| K01953 | asnB, ASNS [EC:6.3.5.4] | AAM | ko00250 | - | + | - | - |
| K01955 | carB, CPA2 [EC:6.3.5.5] | AAM | ko00250 | - | + | - | + |
| K01956 | carA, CPA1 [EC:6.3.5.5] | AAM | ko00250 | + | + | + | + |
| K02437 | gcvH, GCSH | AAM | ko00260 | - | + | - | + |
| K05822 | dapH, dapD [EC:2.3.1.89] | AAM | ko00300 | + | - | + | - |
| K05823 | E3.5.1.47 [EC:3.5.1.47] | AAM | ko00300 | - | + | - | + |
| K06209 | pheB [EC:5.4.99.5] | AAM | ko00400 | + | - | + | - |
| K14155 | patB, malY [EC:4.4.1.8] | AAM | ko00270 | - | + | - | + |
| K15634 | gpmB [EC:5.4.2.12] | AAM | ko00260 | + | + | + | + |
| K05349 | bglX [EC:3.2.1.21] | BSM | ko00940 | - | + | - | + |
| K00215 | dapB [EC:1.17.1.8] | BSM | ko00261 | + | - | + | - |
| K01092 | E3.1.3.25, IMPA, suhB [EC:3.1.3.25] | BSM | ko00521 | + | - | - | - |
| K00928 | lysC [EC:2.7.2.4] | BSM | ko00261 | - | + | + | + |
| K00147 | proA [EC:1.2.1.41] | BSM | ko00332 | - | + | - | + |
| K00931 | proB [EC:2.7.2.11] | BSM | ko00332 | + | - | + | - |
| K01790 | rfbC, rmlC [EC:5.1.3.13] | BSM | ko00521 | - | - | - | + |
| K00067 | rfbD, rmlD [EC:1.1.1.133] | BSM | ko00521 | - | + | - | + |
| K01195 | uidA, GUSB [EC:3.2.1.31] | BSM | ko00944 | - | + | - | + |
| K00027 | ME2, sfcA, maeA [EC:1.1.1.38] | CM | ko00620 | + | - | + | - |
| K00033 | PGD, gnd, gntZ [EC:1.1.1.44 1.1.1.343] | CM | ko00030 | - | + | - | + |
| K00034 | gdh [EC:1.1.1.47] | CM | ko00030 | - | - | + | - |
| K00036 | G6PD, zwf [EC:1.1.1.49 1.1.1.363] | CM | ko00030 | - | + | - | - |
| K00065 | kduD [EC:1.1.1.125] | CM | ko00040 | + | - | + | - |
| K00135 | gabD [EC:1.2.1.16 1.2.1.79 1.2.1.20] | CM | ko00650 | - | + | - | + |
| K00162 | PDHB, pdhB [EC:1.2.4.1] | CM | ko00010,ko00020,  ko00620 | - | + | - | + |
| K00244 | frdA [EC:1.3.5.4] | CM | ko00020,ko00620,  ko00650 | - | + | + | + |
| K00600 | glyA, SHMT [EC:2.1.2.1] | CM | ko00630 | - | + | - | + |
| K00615 | E2.2.1.1, tktA, tktB [EC:2.2.1.1] | CM | ko00030 | + | - | + | - |
| K00625 | E2.3.1.8, pta [EC:2.3.1.8] | CM | ko00620,ko00640 | - | + | - | + |
| K00626 | E2.3.1.9, atoB [EC:2.3.1.9] | CM | ko00620,ko00630,  ko00640,ko00650 | - | + | - | + |
| K00627 | DLAT, aceF, pdhC [EC:2.3.1.12] | CM | ko00010,ko00020,  ko00620 | + | - | + | - |
| K00691 | E2.4.1.8, mapA [EC:2.4.1.8] | CM | ko00500 | - | + | - | + |
| K00790 | murA [EC:2.5.1.7] | CM | ko00520 | - | + | - | - |
| K00851 | E2.7.1.12, gntK, idnK [EC:2.7.1.12] | CM | ko00030 | - | + | - | + |
| K00852 | rbsK, RBKS [EC:2.7.1.15] | CM | ko00030 | - | + | - | + |
| K00865 | glxK, garK [EC:2.7.1.165] | CM | ko00630 | + | + | - | + |
| K00874 | kdgK [EC:2.7.1.45] | CM | ko00030 | + | - | - | - |
| K00925 | ackA [EC:2.7.2.1] | CM | ko00620,ko00640 | + | + | + | + |
| K00948 | PRPS, prsA [EC:2.7.6.1] | CM | ko00030 | + | + | + | + |
| K00963 | UGP2, galU, galF [EC:2.7.7.9] | CM | ko00040,ko00052,  ko00500,ko00520 | - | + | - | + |
| K00965 | galT, GALT [EC:2.7.7.12] | CM | ko00052,ko00520 | - | + | - | + |
| K01091 | gph [EC:3.1.3.18] | CM | ko00630 | + | - | + | - |
| K01092 | E3.1.3.25, IMPA, suhB [EC:3.1.3.25] | CM | ko00562 | + | - | - | - |
| K01182 | IMA [EC:3.2.1.10] | CM | ko00052,ko00500 | - | + | - | + |
| K01187 | malZ [EC:3.2.1.20] | CM | ko00052,ko00500 | - | + | - | + |
| K01190 | lacZ [EC:3.2.1.23] | CM | ko00052 | - | + | + | - |
| K01193 | E3.2.1.26, sacA [EC:3.2.1.26] | CM | ko00052,ko00500 | - | + | - | + |
| K01195 | uidA, GUSB [EC:3.2.1.31] | CM | ko00040,ko00500 | - | + | - | + |
| K01198 | xynB [EC:3.2.1.37] | CM | ko00500,ko00520 | - | + | - | + |
| K01207 | nagZ [EC:3.2.1.52] | CM | ko00520 | - | + | - | + |
| K01208 | E3.2.1.54 [EC:3.2.1.54] | CM | ko00500 | - | + | - | + |
| K01209 | abfA [EC:3.2.1.55] | CM | ko00520 | - | + | - | + |
| K01223 | E3.2.1.86B, bglA [EC:3.2.1.86] | CM | ko00010 | + | + | + | + |
| K01512 | acyP [EC:3.6.1.7] | CM | ko00620 | + | - | + | - |
| K01575 | alsD, budA, aldC [EC:4.1.1.5] | CM | ko00650,ko00660 | - | + | - | + |
| K01624 | FBA, fbaA [EC:4.1.2.13] | CM | ko00010,ko00030,  ko00051 | - | + | - | + |
| K01625 | eda [EC:4.1.2.14 4.1.3.42] | CM | ko00030,ko00630 | + | - | + | - |
| K01641 | E2.3.3.10 [EC:2.3.3.10] | CM | ko00650 | - | + | - | + |
| K01652 | E2.2.1.6L, ilvB, ilvG, ilvI [EC:2.2.1.6] | CM | ko00650,ko00660 | - | + | - | + |
| K01759 | GLO1, gloA [EC:4.4.1.5] | CM | ko00620 | + | - | + | - |
| K01783 | rpe, RPE [EC:5.1.3.1] | CM | ko00030,ko00040 | - | + | - | + |
| K01784 | galE, GALE [EC:5.1.3.2] | CM | ko00052,ko00520 | - | + | - | + |
| K01785 | galM, GALM [EC:5.1.3.3] | CM | ko00010,ko00052 | + | + | + | + |
| K01786 | araD [EC:5.1.3.4] | CM | ko00040 | + | + | - | + |
| K01803 | TPI, tpiA [EC:5.3.1.1] | CM | ko00010,ko00051,  ko00562 | - | + | - | + |
| K01804 | araA [EC:5.3.1.4] | CM | ko00040 | + | - | + | - |
| K01805 | xylA [EC:5.3.1.5] | CM | ko00040,ko00051 | - | + | - | - |
| K01807 | rpiA [EC:5.3.1.6] | CM | ko00030 | + | - | + | - |
| K01834 | PGAM, gpmA [EC:5.4.2.11] | CM | ko00010 | + | - | + | - |
| K01838 | pgmB [EC:5.4.2.6] | CM | ko00500 | - | + | - | + |
| K01854 | glf [EC:5.4.99.9] | CM | ko00052,ko00520 | - | + | - | + |
| K02437 | gcvH, GCSH | CM | ko00630 | - | + | - | + |
| K02564 | nagB, GNPDA [EC:3.5.99.6] | CM | ko00520 | - | + | - | + |
| K02768 | PTS-Fru-EIIA, fruB [EC:2.7.1.202] | CM | ko00051 | - | + | + | + |
| K02769 | PTS-Fru-EIIB, fruA [EC:2.7.1.202] | CM | ko00051 | + | + | + | + |
| K02770 | PTS-Fru-EIIC, fruA | CM | ko00051 | - | + | + | + |
| K02796 | PTS-Man-EIID, manZ | CM | ko00051,ko00520 | + | - | + | - |
| K02802 | PTS-Nag-EIIA, nagE [EC:2.7.1.193] | CM | ko00520 | - | + | - | + |
| K02803 | PTS-Nag-EIIB, nagE [EC:2.7.1.193] | CM | ko00520 | - | + | - | + |
| K02804 | PTS-Nag-EIIC, nagE | CM | ko00520 | - | + | - | + |
| K02809 | PTS-Scr-EIIB, scrA, sacP, sacX, ptsS [EC:2.7.1.-] | CM | ko00500 | - | + | - | - |
| K02810 | PTS-Scr-EIIC, scrA, sacP, sacX, ptsS | CM | ko00500 | - | + | - | - |
| K02818 | PTS-Tre-EIIB, treB [EC:2.7.1.201] | CM | ko00500 | + | - | + | - |
| K02819 | PTS-Tre-EIIC, treB | CM | ko00500 | + | - | + | - |
| K02822 | PTS-Ula-EIIB, ulaB, sgaB [EC:2.7.1.194] | CM | ko00053 | + | - | + | - |
| K03431 | glmM [EC:5.4.2.10] | CM | ko00520 | - | + | - | + |
| K03475 | PTS-Ula-EIIC, ulaA, sgaT | CM | ko00053 | - | - | - | + |
| K03476 | ulaG [EC:3.1.1.-] | CM | ko00053 | - | - | - | + |
| K03778 | ldhA [EC:1.1.1.28] | CM | ko00620 | - | + | - | + |
| K04041 | fbp3 [EC:3.1.3.11] | CM | ko00010,ko00030,  ko00051 | + | - | + | - |
| K05349 | bglX [EC:3.2.1.21] | CM | ko00500 | - | + | - | + |
| K07106 | murQ [EC:4.2.1.126] | CM | ko00520 | + | - | + | - |
| K07404 | pgl [EC:3.1.1.31] | CM | ko00030 | - | + | - | + |
| K07407 | E3.2.1.22B, galA, rafA [EC:3.2.1.22] | CM | ko00052 | + | - | + | - |
| K08093 | hxlA [EC:4.1.2.43] | CM | ko00030 | + | - | + | - |
| K08094 | hxlB [EC:5.3.1.27] | CM | ko00030 | + | - | + | - |
| K12308 | bgaB, lacA [EC:3.2.1.23] | CM | ko00052 | - | + | - | + |
| K15634 | gpmB [EC:5.4.2.12] | CM | ko00010 | + | + | + | + |
| K20116 | PTS-Glc1-EIIA, ptsG, glcA, glcB [EC:2.7.1.199] | CM | ko00010,ko00520 | + | - | + | - |
| K20117 | PTS-Glc1-EIIB, ptsG, glcA, glcB [EC:2.7.1.199] | CM | ko00010,ko00520 | + | - | + | - |
| K20118 | PTS-Glc1-EIIC, ptsG, glcA, glcB | CM | ko00010,ko00520 | + | - | + | - |
| K00925 | ackA [EC:2.7.2.1] | EM | ko00680,ko00720 | + | + | + | + |
| K00058 | serA, PHGDH [EC:1.1.1.95] | EM | ko00680 | - | + | - | + |
| K00244 | frdA [EC:1.3.5.4] | EM | ko00190,ko00720 | - | + | + | + |
| K00262 | E1.4.1.4, gdhA [EC:1.4.1.4] | EM | ko00910 | + | - | + | - |
| K00425 | cydA [EC:1.10.3.14] | EM | ko00190 | - | + | - | + |
| K00426 | cydB [EC:1.10.3.14] | EM | ko00190 | - | + | - | + |
| K00600 | glyA, SHMT [EC:2.1.2.1] | EM | ko00680 | - | + | - | + |
| K00615 | E2.2.1.1, tktA, tktB [EC:2.2.1.1] | EM | ko00710 | + | - | + | - |
| K00625 | E2.3.1.8, pta [EC:2.3.1.8] | EM | ko00680,ko00720 | - | + | - | + |
| K00626 | E2.3.1.9, atoB [EC:2.3.1.9] | EM | ko00720 | - | + | - | + |
| K00651 | metA [EC:2.3.1.46] | EM | ko00920 | + | + | + | - |
| K00937 | ppk [EC:2.7.4.1] | EM | ko00190 | - | - | - | + |
| K01624 | FBA, fbaA [EC:4.1.2.13] | EM | ko00680,ko00710 | - | + | - | + |
| K01738 | cysK [EC:2.5.1.47] | EM | ko00920 | - | + | - | + |
| K01783 | rpe, RPE [EC:5.1.3.1] | EM | ko00710 | - | + | - | + |
| K01803 | TPI, tpiA [EC:5.3.1.1] | EM | ko00710 | - | + | - | + |
| K01807 | rpiA [EC:5.3.1.6] | EM | ko00710 | + | - | + | - |
| K01834 | PGAM, gpmA [EC:5.4.2.11] | EM | ko00680 | + | - | + | - |
| K02109 | ATPF0B, atpF | EM | ko00190,ko00195 | + | - | + | - |
| K02113 | ATPF1D, atpH | EM | ko00190,ko00195 | + | - | + | - |
| K02114 | ATPF1E, atpC | EM | ko00190,ko00195 | + | - | - | + |
| K02115 | ATPF1G, atpG | EM | ko00190,ko00195 | - | + | - | + |
| K02276 | coxC [EC:1.9.3.1] | EM | ko00190 | - | - | + | - |
| K04041 | fbp3 [EC:3.1.3.11] | EM | ko00680,ko00710 | + | - | + | - |
| K06881 | nrnA [EC:3.1.3.7 3.1.13.3] | EM | ko00920 | - | + | - | + |
| K08093 | hxlA [EC:4.1.2.43] | EM | ko00680 | + | - | + | - |
| K08094 | hxlB [EC:5.3.1.27] | EM | ko00680 | + | - | + | - |
| K15552 | tauC | EM | ko00920 | + | - | + | - |
| K15553 | ssuA | EM | ko00920 | + | - | - | + |
| K15554 | ssuC | EM | ko00920 | - | + | - | + |
| K15555 | ssuB [EC:3.6.3.-] | EM | ko00920 | + | - | - | - |
| K15634 | gpmB [EC:5.4.2.12] | EM | ko00680 | + | + | + | + |
| K16937 | doxD [EC:1.8.5.2] | EM | ko00920 | + | - | + | - |
| K00790 | murA [EC:2.5.1.7] | GBM | ko00550 | - | + | - | - |
| K01190 | lacZ [EC:3.2.1.23] | GBM | ko00511 | - | + | + | - |
| K01191 | E3.2.1.24 [EC:3.2.1.24] | GBM | ko00511 | - | + | - | + |
| K01195 | uidA, GUSB [EC:3.2.1.31] | GBM | ko00531 | - | + | - | + |
| K01197 | hya [EC:3.2.1.35] | GBM | ko00531 | - | + | - | + |
| K01207 | nagZ [EC:3.2.1.52] | GBM | ko00531 | - | + | - | + |
| K01921 | ddl [EC:6.3.2.4] | GBM | ko00550 | + | - | + | - |
| K01924 | murC [EC:6.3.2.8] | GBM | ko00550 | - | + | - | + |
| K01925 | murD [EC:6.3.2.9] | GBM | ko00550 | - | + | - | + |
| K01928 | murE [EC:6.3.2.13] | GBM | ko00550 | - | + | - | + |
| K01929 | murF [EC:6.3.2.10] | GBM | ko00550 | - | + | - | + |
| K02563 | murG [EC:2.4.1.227] | GBM | ko00550 | - | + | - | + |
| K03693 | pbp | GBM | ko00550 | - | + | - | + |
| K05366 | mrcA [EC:2.4.1.- 3.4.-.-] | GBM | ko00550 | + | - | + | - |
| K06041 | kdsD, kpsF [EC:5.3.1.13] | GBM | ko00540 | - | - | - | + |
| K07258 | dacC, dacA, dacD [EC:3.4.16.4] | GBM | ko00550 | - | + | - | + |
| K07407 | E3.2.1.22B, galA, rafA [EC:3.2.1.22] | GBM | ko00603 | + | - | + | - |
| K08724 | pbpB | GBM | ko00550 | - | - | - | + |
| K12555 | pbp2A [EC:2.4.1.129 2.3.2.-] | GBM | ko00550 | - | + | - | + |
| K19302 | bcrC [EC:3.6.1.27] | GBM | ko00550 | - | + | - | + |
| K00059 | fabG [EC:1.1.1.100] | LM | ko00061,ko01040 | + | - | + | - |
| K00111 | glpA, glpD [EC:1.1.5.3] | LM | ko00564 | - | + | - | + |
| K00432 | gpx [EC:1.11.1.9] | LM | ko00590 | + | - | + | - |
| K00626 | E2.3.1.9, atoB [EC:2.3.1.9] | LM | ko00071,ko00072 | - | + | - | + |
| K00655 | plsC [EC:2.3.1.51] | LM | ko00561,ko00564 | + | - | + | - |
| K00864 | glpK, GK [EC:2.7.1.30] | LM | ko00561 | - | + | - | + |
| K00865 | glxK, garK [EC:2.7.1.165] | LM | ko00561 | + | + | - | + |
| K00981 | E2.7.7.41, CDS1, CDS2, cdsA [EC:2.7.7.41] | LM | ko00564 | - | + | + | - |
| K00995 | pgsA, PGS1 [EC:2.7.8.5] | LM | ko00564 | + | - | + | - |
| K01126 | E3.1.4.46, glpQ, ugpQ [EC:3.1.4.46] | LM | ko00564 | + | + | + | + |
| K01190 | lacZ [EC:3.2.1.23] | LM | ko00600 | - | + | + | - |
| K01442 | E3.5.1.24 [EC:3.5.1.24] | LM | ko00120,ko00121 | + | - | - | + |
| K01641 | E2.3.3.10 [EC:2.3.3.10] | LM | ko00072 | - | + | - | + |
| K05879 | dhaL [EC:2.7.1.-] | LM | ko00561 | + | - | + | - |
| K06131 | clsA_B [EC:2.7.8.-] | LM | ko00564 | - | + | - | + |
| K07407 | E3.2.1.22B, galA, rafA [EC:3.2.1.22] | LM | ko00561,ko00600 | + | - | + | - |
| K08591 | plsY [EC:2.3.1.15] | LM | ko00561,ko00564 | + | - | + | - |
| K19005 | ltaS [EC:2.7.8.20] | LM | ko00561 | - | + | - | + |
| K00033 | PGD, gnd, gntZ [EC:1.1.1.44 1.1.1.343] | MAA | ko00480 | - | + | - | + |
| K00036 | G6PD, zwf [EC:1.1.1.49 1.1.1.363] | MAA | ko00480 | - | + | - | - |
| K00259 | ald [EC:1.4.1.1] | MAA | ko00430 | - | + | - | + |
| K00383 | GSR, gor [EC:1.8.1.7] | MAA | ko00480 | - | + | - | + |
| K00384 | trxB [EC:1.8.1.9] | MAA | ko00450 | + | - | + | - |
| K00432 | gpx [EC:1.11.1.9] | MAA | ko00480 | + | - | + | - |
| K00600 | glyA, SHMT [EC:2.1.2.1] | MAA | ko00460 | - | + | - | + |
| K00625 | E2.3.1.8, pta [EC:2.3.1.8] | MAA | ko00430 | - | + | - | + |
| K00925 | ackA [EC:2.7.2.1] | MAA | ko00430 | + | + | + | + |
| K01042 | selA [EC:2.9.1.1] | MAA | ko00450 | + | - | + | - |
| K01581 | E4.1.1.17, ODC1, speC, speF [EC:4.1.1.17] | MAA | ko00480 | - | - | - | + |
| K01775 | alr [EC:5.1.1.1] | MAA | ko00473 | - | + | - | + |
| K01776 | murI [EC:5.1.1.3] | MAA | ko00471 | - | + | - | + |
| K01921 | ddl [EC:6.3.2.4] | MAA | ko00473 | + | - | + | - |
| K01924 | murC [EC:6.3.2.8] | MAA | ko00471 | - | + | - | + |
| K01925 | murD [EC:6.3.2.9] | MAA | ko00471 | - | + | - | + |
| K03367 | dltA [EC:6.1.1.13] | MAA | ko00473 | - | + | - | + |
| K03823 | pat [EC:2.3.1.183] | MAA | ko00440 | - | + | - | + |
| K05306 | phnX [EC:3.11.1.1] | MAA | ko00440 | - | + | - | + |
| K05349 | bglX [EC:3.2.1.21] | MAA | ko00460 | - | + | - | + |
| K14155 | patB, malY [EC:4.4.1.8] | MAA | ko00450 | - | + | - | + |
| K00059 | fabG [EC:1.1.1.100] | MCV | ko00780 | + | - | + | - |
| K00135 | gabD [EC:1.2.1.16 1.2.1.79 1.2.1.20] | MCV | ko00760 | - | + | - | + |
| K00287 | folA [EC:1.5.1.3] | MCV | ko00670,ko00790 | + | - | + | - |
| K00560 | thyA, TYMS [EC:2.1.1.45] | MCV | ko00670 | - | + | - | + |
| K00600 | glyA, SHMT [EC:2.1.2.1] | MCV | ko00670 | - | + | - | + |
| K00602 | purH [EC:2.1.2.3 3.5.4.10] | MCV | ko00670 | - | + | - | + |
| K00604 | MTFMT, fmt [EC:2.1.2.9] | MCV | ko00670 | - | + | - | + |
| K00793 | ribE, RIB5 [EC:2.5.1.9] | MCV | ko00740 | - | + | - | + |
| K00794 | ribH, RIB4 [EC:2.5.1.78] | MCV | ko00740 | + | - | + | - |
| K00798 | MMAB, pduO [EC:2.5.1.17] | MCV | ko00860 | - | + | - | + |
| K00826 | E2.6.1.42, ilvE [EC:2.6.1.42] | MCV | ko00770 | + | - | + | - |
| K00858 | ppnK, NADK [EC:2.7.1.23] | MCV | ko00760 | + | - | + | - |
| K00859 | coaE [EC:2.7.1.24] | MCV | ko00770 | + | - | + | - |
| K00867 | coaA [EC:2.7.1.33] | MCV | ko00770 | - | + | - | - |
| K00941 | thiD [EC:2.7.1.49 2.7.4.7] | MCV | ko00730 | + | + | + | + |
| K00949 | thiN, TPK1, THI80 [EC:2.7.6.2] | MCV | ko00730 | + | - | + | - |
| K00954 | E2.7.7.3A, coaD, kdtB [EC:2.7.7.3] | MCV | ko00770 | + | - | + | - |
| K00969 | nadD [EC:2.7.7.18] | MCV | ko00760 | + | - | + | - |
| K00997 | acpS [EC:2.7.8.7] | MCV | ko00770 | + | - | + | - |
| K01195 | uidA, GUSB [EC:3.2.1.31] | MCV | ko00860 | - | + | - | + |
| K01652 | E2.2.1.6L, ilvB, ilvG, ilvI [EC:2.2.1.6] | MCV | ko00770 | - | + | - | + |
| K01662 | dxs [EC:2.2.1.7] | MCV | ko00730 | + | + | - | + |
| K01737 | queD, ptpS, PTS [EC:4.2.3.12 4.1.2.50] | MCV | ko00790 | - | - | + | - |
| K01911 | menE [EC:6.2.1.26] | MCV | ko00130 | + | - | + | - |
| K01916 | nadE [EC:6.3.1.5] | MCV | ko00760 | - | + | - | + |
| K01934 | MTHFS [EC:6.3.3.2] | MCV | ko00670 | + | - | + | - |
| K02548 | menA [EC:2.5.1.74 2.5.1.-] | MCV | ko00130 | + | - | + | - |
| K03183 | ubiE [EC:2.1.1.163 2.1.1.201] | MCV | ko00130 | - | + | - | + |
| K03742 | pncC [EC:3.5.1.42] | MCV | ko00760 | + | - | + | - |
| K03800 | lplA, lplJ [EC:6.3.1.20] | MCV | ko00785 | + | + | + | + |
| K04487 | iscS, NFS1 [EC:2.8.1.7] | MCV | ko00730 | - | + | - | + |
| K11175 | purN [EC:2.1.2.2] | MCV | ko00670 | - | + | - | + |
| K11752 | ribD [EC:3.5.4.26 1.1.1.193] | MCV | ko00740 | + | - | + | - |
| K11753 | ribF [EC:2.7.1.26 2.7.7.2] | MCV | ko00740 | - | + | - | + |
| K11754 | folC [EC:6.3.2.12 6.3.2.17] | MCV | ko00790 | - | + | - | + |
| K13038 | coaBC, dfp [EC:4.1.1.36 6.3.2.5] | MCV | ko00770 | - | + | - | + |
| K14652 | ribBA [EC:4.1.99.12 3.5.4.25] | MCV | ko00740 | + | - | - | - |
| K19267 | qorB [EC:1.6.5.2] | MCV | ko00130 | - | + | - | + |
| K00054 | mvaA [EC:1.1.1.88] | MTP | ko00900 | - | + | - | + |
| K00067 | rfbD, rmlD [EC:1.1.1.133] | MTP | ko00523 | - | + | - | + |
| K00615 | E2.2.1.1, tktA, tktB [EC:2.2.1.1] | MTP | ko01051 | + | - | + | - |
| K00626 | E2.3.1.9, atoB [EC:2.3.1.9] | MTP | ko00900 | - | + | - | + |
| K00791 | miaA, TRIT1 [EC:2.5.1.75] | MTP | ko00908 | - | + | - | + |
| K00806 | uppS [EC:2.5.1.31] | MTP | ko00900 | + | - | + | - |
| K01597 | MVD, mvaD [EC:4.1.1.33] | MTP | ko00900 | + | - | + | - |
| K01641 | E2.3.3.10 [EC:2.3.3.10] | MTP | ko00900 | - | + | - | + |
| K01662 | dxs [EC:2.2.1.7] | MTP | ko00900 | + | + | - | + |
| K01779 | racD [EC:5.1.1.13] | MTP | ko01054 | + | - | + | - |
| K01790 | rfbC, rmlC [EC:5.1.3.13] | MTP | ko00523 | - | - | - | + |
| K01823 | idi, IDI [EC:5.3.3.2] | MTP | ko00900 | - | + | - | + |
| K13789 | GGPS [EC:2.5.1.1 2.5.1.10 2.5.1.29] | MTP | ko00900 | - | + | - | + |
| K00254 | DHODH, pyrD [EC:1.3.5.2] | NM | ko00240 | - | + | - | + |
| K00384 | trxB [EC:1.8.1.9] | NM | ko00240 | + | - | + | - |
| K00525 | E1.17.4.1A, nrdA, nrdE [EC:1.17.4.1] | NM | ko00230,ko00240 | - | + | - | + |
| K00560 | thyA, TYMS [EC:2.1.1.45] | NM | ko00240 | - | + | - | + |
| K00602 | purH [EC:2.1.2.3 3.5.4.10] | NM | ko00230 | - | + | - | + |
| K00609 | pyrB, PYR2 [EC:2.1.3.2] | NM | ko00240 | - | + | - | - |
| K00759 | APRT, apt [EC:2.4.2.7] | NM | ko00230 | + | - | + | - |
| K00760 | hprT, hpt, HPRT1 [EC:2.4.2.8] | NM | ko00230 | + | - | + | - |
| K00762 | pyrE [EC:2.4.2.10] | NM | ko00240 | - | + | - | + |
| K00764 | purF, PPAT [EC:2.4.2.14] | NM | ko00230 | + | - | + | - |
| K00857 | tdk, TK [EC:2.7.1.21] | NM | ko00240 | + | - | + | - |
| K00939 | adk, AK [EC:2.7.4.3] | NM | ko00230 | + | - | + | - |
| K00942 | E2.7.4.8, gmk [EC:2.7.4.8] | NM | ko00230 | + | - | + | - |
| K00943 | tmk, DTYMK [EC:2.7.4.9] | NM | ko00240 | + | - | + | - |
| K00945 | cmk [EC:2.7.4.14] | NM | ko00240 | + | - | - | - |
| K00948 | PRPS, prsA [EC:2.7.6.1] | NM | ko00230 | + | + | + | + |
| K01465 | URA4, pyrC [EC:3.5.2.3] | NM | ko00240 | + | + | + | + |
| K01485 | codA [EC:3.5.4.1] | NM | ko00240 | - | - | + | - |
| K01487 | E3.5.4.3, guaD [EC:3.5.4.3] | NM | ko00230 | - | + | - | + |
| K01489 | cdd, CDA [EC:3.5.4.5] | NM | ko00240 | + | - | + | - |
| K01515 | nudF [EC:3.6.1.13] | NM | ko00230 | + | - | + | - |
| K01520 | dut, DUT [EC:3.6.1.23] | NM | ko00240 | + | - | + | - |
| K01524 | ppx-gppA [EC:3.6.1.11 3.6.1.40] | NM | ko00230 | - | + | - | + |
| K01588 | purE [EC:5.4.99.18] | NM | ko00230 | + | - | + | - |
| K01589 | purK [EC:6.3.4.18] | NM | ko00230 | + | + | + | + |
| K01591 | pyrF [EC:4.1.1.23] | NM | ko00240 | - | + | - | + |
| K01756 | purB, ADSL [EC:4.3.2.2] | NM | ko00230 | - | + | - | + |
| K01933 | purM [EC:6.3.3.1] | NM | ko00230 | - | + | - | + |
| K01939 | purA, ADSS [EC:6.3.4.4] | NM | ko00230 | - | + | - | + |
| K01945 | purD [EC:6.3.4.13] | NM | ko00230 | - | + | + | - |
| K01951 | guaA, GMPS [EC:6.3.5.2] | NM | ko00230 | - | + | - | + |
| K01952 | purL, PFAS [EC:6.3.5.3] | NM | ko00230 | + | + | + | + |
| K01955 | carB, CPA2 [EC:6.3.5.5] | NM | ko00240 | - | + | - | + |
| K01956 | carA, CPA1 [EC:6.3.5.5] | NM | ko00240 | + | + | + | + |
| K02335 | DPO1, polA [EC:2.7.7.7] | NM | ko00230,ko00240 | - | + | - | + |
| K02337 | DPO3A1, dnaE [EC:2.7.7.7] | NM | ko00230,ko00240 | - | + | - | + |
| K02338 | DPO3B, dnaN [EC:2.7.7.7] | NM | ko00230,ko00240 | + | - | + | - |
| K02340 | DPO3D1, holA [EC:2.7.7.7] | NM | ko00230,ko00240 | - | + | - | + |
| K02341 | DPO3D2, holB [EC:2.7.7.7] | NM | ko00230,ko00240 | - | + | - | + |
| K02342 | DPO3E, dnaQ [EC:2.7.7.7] | NM | ko00230,ko00240 | + | - | + | - |
| K02343 | DPO3G, dnaX [EC:2.7.7.7] | NM | ko00230,ko00240 | + | - | + | - |
| K02825 | pyrR [EC:2.4.2.9] | NM | ko00240 | + | - | + | - |
| K03043 | rpoB [EC:2.7.7.6] | NM | ko00230,ko00240 | + | - | - | - |
| K03046 | rpoC [EC:2.7.7.6] | NM | ko00230,ko00240 | - | + | - | + |
| K03048 | rpoE | NM | ko00230,ko00240 | + | - | + | - |
| K03060 | rpoZ [EC:2.7.7.6] | NM | ko00230,ko00240 | + | - | + | - |
| K07816 | E2.7.6.5X [EC:2.7.6.5] | NM | ko00230 | + | - | + | - |
| K09903 | pyrH [EC:2.7.4.22] | NM | ko00240 | - | - | - | + |
| K11175 | purN [EC:2.1.2.2] | NM | ko00230 | - | + | - | + |
| K00014 | aroE [EC:1.1.1.25] | OV | ko01230 | + | + | + | + |
| K00027 | ME2, sfcA, maeA [EC:1.1.1.38] | OV | ko01200 | + | - | + | - |
| K00033 | PGD, gnd, gntZ [EC:1.1.1.44 1.1.1.343] | OV | ko01200 | - | + | - | + |
| K00034 | gdh [EC:1.1.1.47] | OV | ko01200 | - | - | + | - |
| K00036 | G6PD, zwf [EC:1.1.1.49 1.1.1.363] | OV | ko01200 | - | + | - | - |
| K00055 | E1.1.1.90 [EC:1.1.1.90] | OV | ko01220 | - | + | - | - |
| K00058 | serA, PHGDH [EC:1.1.1.95] | OV | ko01200,ko01230 | - | + | - | + |
| K00059 | fabG [EC:1.1.1.100] | OV | ko01212 | + | - | + | - |
| K00147 | proA [EC:1.2.1.41] | OV | ko01230 | - | + | - | + |
| K00162 | PDHB, pdhB [EC:1.2.4.1] | OV | ko01200 | - | + | - | + |
| K00215 | dapB [EC:1.17.1.8] | OV | ko01230 | + | - | + | - |
| K00244 | frdA [EC:1.3.5.4] | OV | ko01200 | - | + | + | + |
| K00286 | proC [EC:1.5.1.2] | OV | ko01230 | + | - | + | - |
| K00600 | glyA, SHMT [EC:2.1.2.1] | OV | ko01200,ko01230 | - | + | - | + |
| K00615 | E2.2.1.1, tktA, tktB [EC:2.2.1.1] | OV | ko01200,ko01230 | + | - | + | - |
| K00625 | E2.3.1.8, pta [EC:2.3.1.8] | OV | ko01200 | - | + | - | + |
| K00626 | E2.3.1.9, atoB [EC:2.3.1.9] | OV | ko01200,ko01212 | - | + | - | + |
| K00627 | DLAT, aceF, pdhC [EC:2.3.1.12] | OV | ko01200 | + | - | + | - |
| K00651 | metA [EC:2.3.1.46] | OV | ko01230 | + | + | + | - |
| K00789 | metK [EC:2.5.1.6] | OV | ko01230 | - | - | - | + |
| K00800 | aroA [EC:2.5.1.19] | OV | ko01230 | - | + | - | + |
| K00826 | E2.6.1.42, ilvE [EC:2.6.1.42] | OV | ko01210,ko01230 | + | - | + | - |
| K00841 | patA [EC:2.6.1.-] | OV | ko01230 | - | + | - | + |
| K00851 | E2.7.1.12, gntK, idnK [EC:2.7.1.12] | OV | ko01200 | - | + | - | + |
| K00874 | kdgK [EC:2.7.1.45] | OV | ko01200 | + | - | - | - |
| K00925 | ackA [EC:2.7.2.1] | OV | ko01200 | + | + | + | + |
| K00928 | lysC [EC:2.7.2.4] | OV | ko01210,ko01230 | - | + | + | + |
| K00931 | proB [EC:2.7.2.11] | OV | ko01230 | + | - | + | - |
| K00948 | PRPS, prsA [EC:2.7.6.1] | OV | ko01200,ko01230 | + | + | + | + |
| K01243 | mtnN, mtn, pfs [EC:3.2.2.9] | OV | ko01230 | - | + | - | + |
| K01439 | dapE [EC:3.5.1.18] | OV | ko01230 | - | + | - | + |
| K01586 | lysA [EC:4.1.1.20] | OV | ko01230 | + | - | + | - |
| K01607 | pcaC [EC:4.1.1.44] | OV | ko01220 | + | - | + | - |
| K01624 | FBA, fbaA [EC:4.1.2.13] | OV | ko01200,ko01230 | - | + | - | + |
| K01625 | eda [EC:4.1.2.14 4.1.3.42] | OV | ko01200 | + | - | + | - |
| K01652 | E2.2.1.6L, ilvB, ilvG, ilvI [EC:2.2.1.6] | OV | ko01210,ko01230 | - | + | - | + |
| K01738 | cysK [EC:2.5.1.47] | OV | ko01200,ko01230 | - | + | - | + |
| K01783 | rpe, RPE [EC:5.1.3.1] | OV | ko01200,ko01230 | - | + | - | + |
| K01803 | TPI, tpiA [EC:5.3.1.1] | OV | ko01200,ko01230 | - | + | - | + |
| K01807 | rpiA [EC:5.3.1.6] | OV | ko01200,ko01230 | + | - | + | - |
| K01821 | praC, xylH [EC:5.3.2.6] | OV | ko01220 | + | - | + | - |
| K01834 | PGAM, gpmA [EC:5.4.2.11] | OV | ko01200,ko01230 | + | - | + | - |
| K04041 | fbp3 [EC:3.1.3.11] | OV | ko01200 | + | - | + | - |
| K05822 | dapH, dapD [EC:2.3.1.89] | OV | ko01230 | + | - | + | - |
| K05823 | E3.5.1.47 [EC:3.5.1.47] | OV | ko01230 | - | + | - | + |
| K06209 | pheB [EC:5.4.99.5] | OV | ko01230 | + | - | + | - |
| K07404 | pgl [EC:3.1.1.31] | OV | ko01200 | - | + | - | + |
| K08093 | hxlA [EC:4.1.2.43] | OV | ko01200,ko01230 | + | - | + | - |
| K08094 | hxlB [EC:5.3.1.27] | OV | ko01200,ko01230 | + | - | + | - |
| K14155 | patB, malY [EC:4.4.1.8] | OV | ko01230 | - | + | - | + |
| K15634 | gpmB [EC:5.4.2.12] | OV | ko01200,ko01230 | + | + | + | + |
| K00055 | E1.1.1.90 [EC:1.1.1.90] | XBM | ko00622,ko00623 | - | + | - | - |
| K00626 | E2.3.1.9, atoB [EC:2.3.1.9] | XBM | ko00362 | - | + | - | + |
| K00760 | hprT, hpt, HPRT1 [EC:2.4.2.8] | XBM | ko00983 | + | - | + | - |
| K00857 | tdk, TK [EC:2.7.1.21] | XBM | ko00983 | + | - | + | - |
| K01195 | uidA, GUSB [EC:3.2.1.31] | XBM | ko00983 | - | + | - | + |
| K01489 | cdd, CDA [EC:3.5.4.5] | XBM | ko00983 | + | - | + | - |
| K01512 | acyP [EC:3.6.1.7] | XBM | ko00627 | + | - | + | - |
| K01560 | E3.8.1.2 [EC:3.8.1.2] | XBM | ko00361,ko00625 | + | - | + | - |
| K01607 | pcaC [EC:4.1.1.44] | XBM | ko00362 | + | - | + | - |
| K01821 | praC, xylH [EC:5.3.2.6] | XBM | ko00362,ko00621,  ko00622 | + | - | + | - |
| K01951 | guaA, GMPS [EC:6.3.5.2] | XBM | ko00983 | - | + | - | + |

* AAM, amino acid metabolism; BSM, Biosynthesis of other secondary metabolites; CM, carbohydrate metabolism; EM, energy metabolism; GBM, Glycan biosynthesis and metabolism; LM, Lipid metabolism; MAA, Metabolism of other amino acids; MCV, Metabolism of cofactors and vitamins; MTP, Metabolism of terpenoids and polyketides; NM, nucleotide metabolism; OV, overview; XBM, Xenobiotics biodegradation and metabolism.

** LA: *L. alimentarius*; LPA: *L. paralimentarius*; +: detected; -: not detected.
