# Supplementary material for: Dynamic and Functional Characteristics of Predominant Species in Industrial Paocai as Revealed by Combined DGGE and Metagenomic Sequencing
Source: Front Microbiol. 2018 Oct 9;9:2416. doi: 10.3389/fmicb.2018.02416 (PMC6189446; doi:10.3389/fmicb.2018.02416)
Supplement: Supplementary file 3 [file Image_1.pdf]

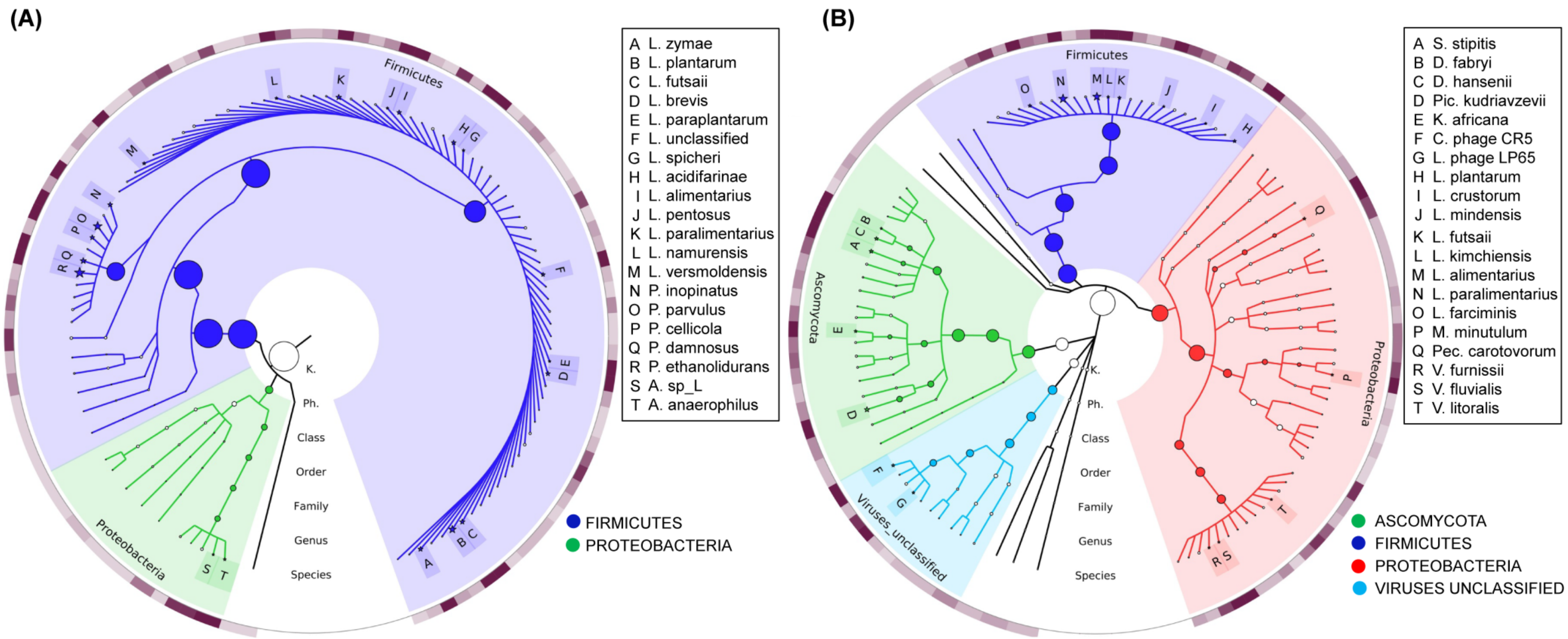

Supplementary Figure S1 Taxonomy of the microbial community in matured industrial ZP (A) and QP (B) using metagenomic sequencing. Only the species with abundance in top 100 was presented and the top 20 were labeled by stars. The size of star and the color thickness of the outside ring represent the abundance of species. L.: *Lactobacillus*, P.: *Pediococcus*, A.: *Arcobacter*, S.: *Scheffersomyces*, D.: *Debaryomyces*, Pic.: *Pichia*, K.: *Kazachstania*, C.: *Cronobacter*, M.: *Marinospirillum*, Pec.: *Pectobacterium*, V.: *Vibrio*.
